# Focusing on Attention: The Effects of Working Memory Capacity and Load on Selective Attention

**DOI:** 10.1371/journal.pone.0043101

**Published:** 2012-08-28

**Authors:** Lubna Ahmed, Jan W. de Fockert

**Affiliations:** Goldsmiths, University of London, London, United Kingdom; Georgia Health Sciences University, United States of America

## Abstract

**Background:**

Working memory (WM) is imperative for effective selective attention. Distractibility is greater under conditions of high (vs. low) concurrent working memory load (WML), and in individuals with low (vs. high) working memory capacity (WMC). In the current experiments, we recorded the flanker task performance of individuals with high and low WMC during low and high WML, to investigate the combined effect of WML and WMC on selective attention.

**Methodology/Principal Findings:**

In Experiment 1, distractibility from a distractor at a fixed distance from the target was greater when either WML was high or WMC was low, but surprisingly smaller when both WML was high and WMC low. Thus we observed an inverted-U relationship between reductions in WM resources and distractibility. In Experiment 2, we mapped the distribution of spatial attention as a function of WMC and WML, by recording distractibility across several target-to-distractor distances. The pattern of distractor effects across the target-to-distractor distances demonstrated that the distribution of the attentional window becomes dispersed as WM resources are limited. The attentional window was more spread out under high compared to low WML, and for low compared to high WMC individuals, and even more so when the two factors co-occurred (i.e., under high WML in low WMC individuals). The inverted-U pattern of distractibility effects in Experiment 1, replicated in Experiment 2, can thus be explained by differences in the spread of the attentional window as a function of WM resource availability.

**Conclusions/Significance:**

The current findings show that limitations in WM resources, due to either WML or individual differences in WMC, affect the spatial distribution of attention. The difference in attentional constraining between high and low WMC individuals demonstrated in the current experiments helps characterise the nature of previously established associations between WMC and controlled attention.

## Introduction

Over the last two decades, research findings demonstrating an association between working memory capacity (WMC) and executive attention capabilities have accumulated [1]. In tasks of visual selective attention, individuals with high WMC are typically more effective at selectively attending to relevant, and overcoming the influence of irrelevant information, compared to individuals with low WMC [2]–[7]. For instance, during the Stroop task, low WMC individuals are more prone to interference from the irrelevant attribute of the stimulus than those with high WMC [2]. Similarly, the interference effects of visual distractors are greater for low, compared to high WMC individuals when performing the Eriksen flanker task [3]. Such findings have led to the suggestion that attentional control mechanisms are more efficient in individuals with greater, compared to those with more limited, working memory (WM) resources.

In parallel, but independent of the findings that individual differences in WMC are predictive of selective attention efficiency, evidence that manipulations of concurrent working memory load (WML) similarly influence visual selective attention efficiency has also built up [8], [9]. Lavie et al. [8] found that the detrimental influence of distractors in the flanker task was greater in conditions of high, compared to low concurrent WML. Similarly, processing of irrelevant information in a Stroop-like task was found to increase as a function of concurrent WML [9]. Load theory of attention [8] proposes that WML depletes limited-capacity cognitive resources that are required to maintain goal distinctions between processed relevant and irrelevant information. Consequently, behaviour becomes more susceptible to be led by irrelevant information when WML is high.

Whereas the influence of reduced WM resources on visual selective attention due to individual differences on the one hand, and imposed load on the other, is well established, the interactive impact on attention of limiting WM availability by these factors has not been investigated previously. Thus, it remains unclear if the effect of additional WML is the same in individuals who vary in terms of WMC, or whether, for example, performance in low WMC individuals is especially impaired when additional WML is imposed. The objective of the current study was to establish how selective attention of individuals that differ in WMC is affected as concurrent WML is increased, by measuring distractibility in individuals with either low or high WMC, under both low and high WML.

Since individuals with low WMC show greater distractor interference effects than those with high WMC [2], [3], and increasing the level of concurrent WML leads to greater interference within individuals [8], [9], our first prediction was of an additive effect of WML and WMC on distractibility. On this view, distractor effects will be smallest in high WMC individuals under low WML, as this represents the situation in which most WM resources are available for selective attention. Intermediate distractor effects are predicted for high WMC individuals under high WML, as well as for low WMC individuals under low WML. Finally, distractor effects will be greatest in low WMC individuals under high WML, as this represents the situation in which WM is least available for selective attention.

Although the combined effect of WMC and imposed WML has not previously been examined in visual selective attention, previous studies have investigated how memory performance varies as a function of WMC and concurrent cognitive load. These studies suggest that the nature of the interaction between WMC and cognitive load may in fact be different to the additive effect proposed above. For instance, Kane and Engle [10] found that proactive interference in the recall of list items was greater in individuals with low WMC, compared to those with high WMC under single task conditions. When a concurrent finger tapping task was introduced, proactive interference increased in the high WMC group, but remained unchanged in the low WMC group. A similar pattern was reported by Rosen and Engle [11] in a verbal fluency task that required generating category exemplars without repetition, in combination with a concurrent digit-tracking task. In the single task condition, performance was worse in the low, compared to the high WMC group. For the high WMC group, the additional load in the dual task condition led to a reduction in verbal fluency compared to the single task condition, while performance in the low WMC group remained the same across both conditions.

In these studies, the ‘pseudo-resistance’ to cognitive load in low WMC individuals is interpreted to reflect ceiling effects; scores did not deteriorate with load in the low WMC individuals because their performance was already maximally affected during the comparatively lower load single task condition. Our second prediction was therefore that distractibility would increase as WM resources are reduced, due to either imposed WML or WMC limitations, until distractor effects are maximal. If individuals with low WMC already show considerable interference effects under low WML, then, in contrast to the monotonic increase outlined above, distractibility may not become reliably greater when WML is increased.

In the current study, distractibility was measured in the flanker task [12]. Participants were required to respond to a centrally presented target letter while ignoring a peripheral distractor letter, which could be either the same (congruent condition) or different (incongruent condition) to the target letter. When attention is imperfectly restricted to the target letter, the peripheral distractor is also processed, and performance is worse on incongruent trials, compared to congruent trials (i.e. the congruency effect). In Experiment 1, we used a factorial design to measure the congruency effect in individuals with either high or low WMC, and under conditions of low and high WML to investigate the interactive influence of limiting WM resources via WMC and WML on selective attention.

## Experiment 1

### Ethics statement

This project was considered and approved using agreed Departmental procedures by the Chair of the departmental ethics committee at Goldsmiths. Informed written consent was obtained from all participants prior to taking part. The consent form outlined the procedure of the study, maintenance of participant anonymity, the right to withdraw at any point during the study and/or destruction of recorded data, and that they would have an opportunity to ask questions following the study. All participants were debriefed on the study's objective following their participation and any questions answered by the researcher.

### Method

**Participants:** Forty-nine university students (11 males, mean age = 20.39 years, SD = 3.08) took part in the study. All had reported normal or corrected–to-normal vision, and received course credits or payment for taking part.

**WMC screening:** Each participant completed the standardised automated Operation span task (Aospan), developed by Unsworth, Heitz, Schrock, & Engle [13] to measure their WMC. This task requires maintaining a set of letters in memory whilst solving a series of maths equations. Each trial began with the presentation of a maths equation (e.g. ‘(8/2)−1 = ?’). Participants were instructed to mentally solve the equation then click the mouse to proceed. A single digit (e.g. 3) was then presented; participants had to click either the ‘true’ or ‘false’ box to indicate if the digit was the correct answer to the preceding equation. A single letter to be retained for later recall was then presented. Between three and seven equation-letter pairs (set size 3–7) were presented before participants were instructed to recall the letters in the presented order, by selecting them from a screen containing all twelve letters used in the experiment (F, H, J, K, L, N, P, Q, R, S, T, and Y). Each set size was repeated three times, and set presentation order was randomised. In total, participants completed 75 equations and letter strings. Following standard Aospan procedure, participants with equation accuracies below 85% were excluded and the standard absolute scoring method was used, in which the WMC score is the total number of letters correctly recalled in sets in which all letters were correctly recalled (score range 0–75). Three practice blocks (equation only, letter only, and equation and letter together) were completed prior to the task. The Aospan took approximately 20 minutes to complete, and was always completed before the flanker task.

**Figure 1:**
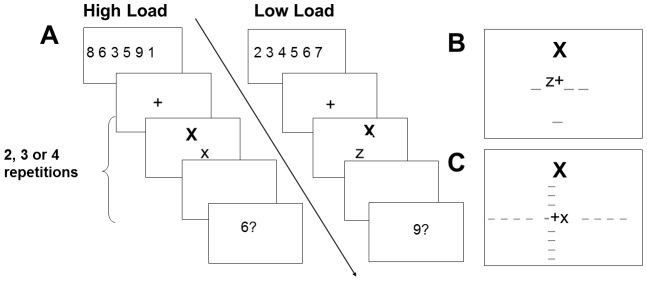
**Experiment 1** **trial sequence and displays.** A) Sample trial sequence for a congruent (left) and incongruent (right) trial, under high load and low WML conditions respectively. B) Sample of the letter identification display in Experiment 1. C) Sample of the letter identification display in Experiment 2. Dashes represent possible target and distractor positions and were not displayed in the experiment.

**Stimuli and procedure:** Participants were tested using E-Prime software (version 1.1. SP3; [14]) in a dimly lit testing cubicle, seated approximately 50 cm from a 17inch monitor. An experimenter was present during the practice block to ensure participants maintained the viewing distance. Responses were collected using a standard keyboard. The flanker trials consisted of an attention (letter identification) and WM (digit recall) component. See Figure 1 for a sample trial sequence. For the memory component, a six digit set consisting of digits between 1 and 9 and subtending a visual angle of 10.5° was displayed in Arial font size 32 in the centre of the screen. In the low WML condition the digits were in sequentially ascending order. In the high WML condition the digits were in random non-sequential order. The digits sets were displayed for 2000 ms followed by a 1500 ms blank screen. Next, the letter identification task was presented (see below for details). At the end of the trial a single digit memory probe was presented for 5000 ms or until response. Participants were instructed to press the ‘w’ key if they thought the probe had been present in the memory set, and the ‘s’ key if they thought it had not, using the middle and index fingers of their left hand, respectively. Key allocations were counterbalanced between participants. The probe digit was equally likely to have been present or absent in the set, and if present, equally likely to have occurred in any of the six memory set positions. A 500 ms blank screen inter-trial interval was presented before the start of the next memory trial.

After presentation of the memory set, and before the memory probe, the letter identification task was presented. In order to ensure that the memory set was actively rehearsed during the entire trial, the presentation of the memory probe was made unpredictable, by varying the number of letter trials [9]. Either two, three or four letter identification trials were presented during each WM trial. Each letter identification trial began with a 500 ms blank screen, followed by a central fixation cross, subtending 0.57° by 0.57°, which was equally likely to be presented for 500, 750 or 1000 ms. The fixation duration was varied to discourage participants from adopting a strategy that involved predicting the onset of the next letter display. The letter display was then presented for 200 ms, containing a target and a distractor letter. Participants had up to 1500 ms to press the ‘0’ key if they thought the centrally presented target letter was a ‘z’, and the ‘2’ key if they thought it was ‘x’ with the middle and index fingers of their right hand, respectively. Key allocations were counterbalanced between participants. A response, or lapse of the response window triggered the next letter identification trial, or the memory probe display. Feedback following incorrect or absent responses during both the memory and letter components was given with a 200 ms screen containing a red cross.

The target letter was presented in lower case Arial Font size 18, and subtended 0.57° by 0.57°. It was equally likely to be a ‘z’ or an ‘x’, and equally likely to appear in one of four positions along an imaginary horizontal line centred at fixation. The possible target positions were equally spaced with an edge-to-edge distance of 0.37° between them. The edge-to-edge distance between the outer left and outer right target positions was approximately 2.26°.

A distractor letter subtending a visual angle of 1.15° by 1.15° was presented simultaneously with the target letter, in upper case Arial Font size 28. The distractor letter was either a ‘X’ or ‘Z’, and was equally likely to appear in one of two possible locations; either directly above or below the position of the central fixation cross. The edge-to-edge target-to-distractor distance was 3.44° for the two central target positions, and 3.60° for the outer target positions (see Figure 1b). The distractor letter was equally likely to be the same (congruent) or different to the target letter (incongruent). The combinations of target identity (2), target position (4), distractor identity (2), and distractor position (2) generated 32 unique displays, each of which was repeated twice within each load block.

Either two, three or four letter identification trials were presented for each WM trial. There were ten WM trials that had two letter identification trials, eight WM trials with three letter identification trials, and five WM trials with four letter identification trials, the order of which was randomised. Each participant completed four experimental blocks. Each block began with one practice trial, followed by 23 WM trials, and a total of 64 letter trials. WML (high or low) was varied between blocks, using two block orders (LHLH and HLHL) which were counterbalanced between participants. Participants were informed of the load type at the start of each block, and break intervals were included between experimental blocks. Participants completed two practice blocks of each load condition, consisting of three load trials and between 6–12 letter trials per load condition. The experimental session lasted 45–55 minutes in total.

## Results

**Data screening:** Data from six participants, whose average accuracy on either the WM or the flanker task was below chance, were removed from the analysis.

**WMC groups:** From the remaining 43 participants, those with Aospan scores in the upper and lower quartiles were classified as high and low WMC individuals, respectively. This resulted in 11 participants per WMC group. (Aospan Score: Low WMC Group: M = 13.27, SD = 6.47; High WMC Group M = 51.45, SD = 8.22).

**WM task:** Accuracy, rather than speed, was emphasised to participants for the memory probe response, and only the probe error rates were analysed. Mean error rate was computed for participants in each WMC group as a function of WML (Table 1). The errors rates were analysed in a 2×2 Analysis of Variance (ANOVA) with WML (low, high) as a within subjects factor and WMC (low, high) as a between subjects factor. Probe error rates were lower under low WML (M = 12%) compared to high WML (M = 19%; F(1,20) = 8.13, MSE = 0.006, p<.010, 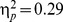), which confirmed that the load manipulation was successful. Neither the main effect of WMC group, nor the two-way interaction were significant (both p>.6), indicating that the level of WML affected memory task performance similarly in the two WMC groups.

**Table 1:** Experiment 1 & 2: Mean correct reaction times (in milliseconds) and error rates on the memory task as a function of WML and WMC group.

	Low WMC		High WMC	
	RT	Error rate	RT	Error rate
**Experiment 1**				
Low WML	1245 (260)	.12 (.08)	1425 (173)	.12 (.08)
High WML	1302 (201)	.20 (.13)	1455 (171)	.18 (.13)
**Experiment 2**				
Low WML	1300 (276)	.16 (.09)	1273 (210)	.13 (.09)
High WML	1295 (289)	.25 (.10)	1225 (224)	.15 (.08)

Values in brackets are Standard Deviations.

**Flanker task:** For the analysis of letter identification responses, trials with responses that were incorrect or faster than 200 ms were excluded. In addition, letter responses were excluded from the analysis if the response to the memory probe for that trial was incorrect. Mean correct RT and error rate were computed for each participant in the two WMC groups as a function of congruency condition and WML (Table 2), and entered into two 2×2×2 ANOVAs, with congruency (congruent, incongruent) and WML (low, high) as within, and WMC Group (low, high) as a between subjects factor.

**Table 2:** Experiment 1: Mean correct reaction times (in milliseconds) and error rates on the letter identification task as a function of WML, congruency type, and WMC groups.

	Low WMC		High WMC	
	RT	Error rate	RT	Error rate
**Low WML**				
Congruent	710 (78)	.08 (.06)	699 (116)	.11 (.09)
Incongruent	754 (111)	.10 (.09)	714 (148)	.14 (.08)
Congruency Effect	45 (51)	.01 (.06)	16 (16)	.03 (.05)
**High WML**				
Congruent	714 (80)	.09 (.06)	694 (118)	.14 (.09)
Incongruent	734 (86)	.13 (.10)	745 (141)	.15 (.12)
Congruency Effect	20 (38)	.04 (.05)	51 (40)	.01 (.08)

Values in brackets are Standard Deviations.

**Figure 2:**
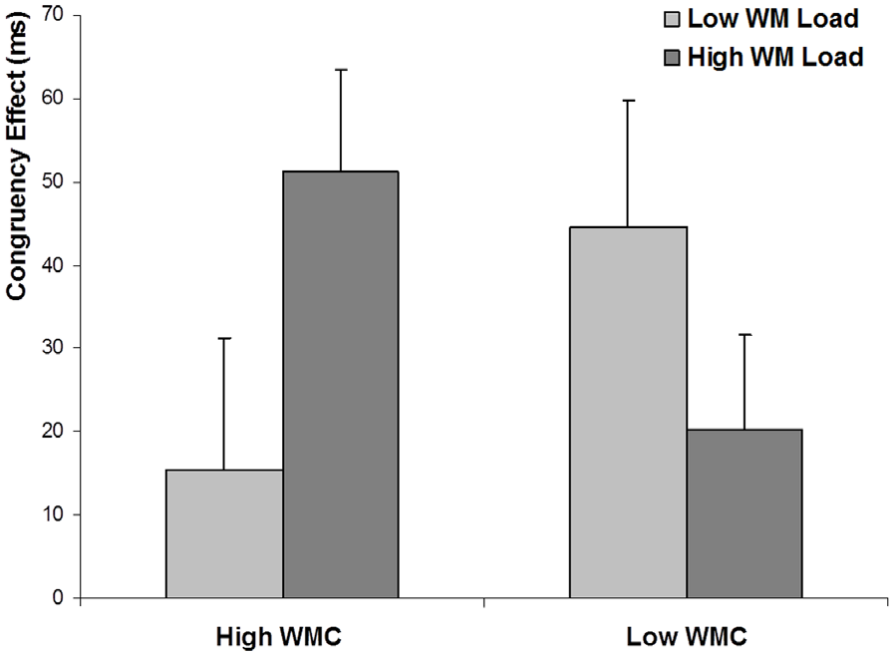
**Experiment 1** **RT congruency effects graph.** Mean RTs congruency effect under low and high WML conditions, for the low and high WMC groups in Experiment 1. Error bars are standard errors.

The RT ANOVA revealed a main effect of congruency, F(1, 20) = 15.204, MSE = 23848.90, p<.001, 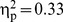; RTs were longer on incongruent (737 ms) compared to congruent trials (704 ms). All remaining main effects and two-way interactions were non-significant (p>.1 in all cases), However, the three-way interaction between congruency, WML, and WMC was reliable, F(1, 20) = 9.84, MSE = 1010.17, p<.005, 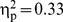. In the high WMC group, there was a greater difference between congruent and incongruent trials (i.e. the congruency effect) when WML was high (51 ms) compared to low (16 ms); t(10) = 3.73, SEM = 9.62, p<.004). For the low WMC group, the congruency effect was smaller during the high (20 ms) versus low WML trials (45 ms), although this effect failed to reach significance (t(10) = 1.47, SEM = 16.58, p>.1) (see Figure 2).

Next, we ran an analysis including all participants, as a further test of the relationship between the factors of WMC and WML, and distractibility. The 21 participants not included in the initial analysis were added to this analysis. They had intermediate WMC (Aospan Score, M = 30.55, SD = 6.1). In order to test if WMC significantly predicted RT congruency effects in the low and high WM load conditions, we ran regression analyses with WMC as a predictor, and congruency effect as the predicted variable. The results of the regression indicated that there was an inverse, but non-significant relationship between WMC and congruency effects under low WML (*β = −.*623; R_2_ = .047, F(1,41) = 2.01, p = .164). Conversely, under high WML there was a positive and significant relationship between WMC and congruency effects (*β = .*983, R_2_ = .129, F(1,41) = 6.09, p = 0.018). This finding again suggests that increasing WML has opposite effects on distractibility in individuals with varying levels of WMC, with a tendency for reduced interference with increasing WMC when WML is low, but greater interference with increasing WMC when WML is high.

Error rates were analysed in the same way as RTs. The 2×2×2 ANOVA on the error rates revealed main effects of congruency, F(1, 20) = 5.58, MSE = .013, p<.029, 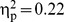 and WML, F(1, 20) = 5.22, MSE = .011, p<.033, 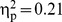. Error rates were on average higher on incongruent (.13) compared to congruent (.11) trials, and under high WML (.13) compared to low WMC (.11) trials. The main effect of WMC and all remaining interactions were non-significant (p>.1 in all cases). Also in the error rate regression analysis including all participants, there was no significant association between WMC and interference in low WML (R_2_ = .0.001, F(1,41) = .002, p = 0.968) and high WML (R_2_ = .033, F(1,41) = 1.40, p = .245).

### Discussion

The objective of Experiment 1 was to investigate the interactive effect of imposed WML and individual differences in WMC on the ability to selectively attend to a target letter in the flanker task. We predicted that, if the level of distractibility depends on the availability of WM resources, then distractibility effects would increase with limitations in WM resources. Thus, distractor effects would be smallest in the high WMC group under low WML, and greater for this group under high WML, as well as for the low WMC group under low WML. The results fully support this part of the prediction. However, in contrast to our prediction, when WM resources were most depleted (i.e. in the low WMC group under high WML), distractor effects were neither greater than, nor as great as they were under low WML. Whereas in the high WMC group, increasing WML had the expected and previously reported effect of increasing the congruency effect [8], [9], we found an unexpected reduction in the congruency effect in the low WMC group when WML was increased. In other words, with reductions in the availability of WM, distractibility effects showed neither a monotonic increase nor a ceiling effect, but an inverted-U shaped function.

Whereas congruency effects were significantly increased with increasing WML in the high WMC group, the reduction in congruency effects with increasing WML in the low WMC group was not statistically reliable. One might therefore conclude that these results support the prediction that distractibility is subject to a ceiling effect, such that increasing WML in the already highly distractible low WMC group does not lead to a further increase in distractibility. We were however surprised by the magnitude of the reduction in the congruency effect as a function of WML in the low WMC group, which was more than halved under high (versus low) WML. To ensure we were not committing a Type II error, we designed Experiment 2 to further investigate the pattern of congruency effects we observed as a function of WMC and WML.

What could account for a reduction in distractibility in the low WMC group under high WML? It is possible that individuals in the low WMC group sacrificed performance in the WM task under high WML in order to maintain performance on the selective attention task. However, WM performance was equally affected by WML in the two WMC groups, indicating that this was not the case. Instead, we propose that the modulation of congruency effects as a function of the availability of WM resources, including the apparent enhancement in the efficiency of selective attention during conditions of extreme depletion (i.e low WMC individuals under high WML), can be explained by considering how the spatial distribution of attention may be affected by limitations in WM.

Traditionally, the distribution of spatial attention is described to decline linearly as the distance from the focus of attention increases [15], [16]. However, contrary to this view, several recent studies have demonstrated that distractor effects can be greater at further compared to closer distances from the attended location [17]–[19]. For example, Muller, Mollenhauer, Rosler, and Kleinschmidt [17] found that interference effects from peripheral distractors were largest at the distance closest to the attended location, (1.3° from the target), declined at 2.5°, but then increased at the further distance of 4.7°. Such a pattern of distractor effects suggests that the distribution of attention does not decrease monotonically from the attended location, but rather that spatial attention follows a non-monotonic profile in the shape of a ‘Mexican-hat’ from the focus of attention. According to the Mexican-hat model, the attentional window is comprised of attention and suppression zones. The central attended zone (first attention zone, a1) is surrounded by a suppression zone (first suppression zone, s1), a second attention zone (a2) and finally a peripheral suppression or unattended zone (s2).

**Figure 3:**
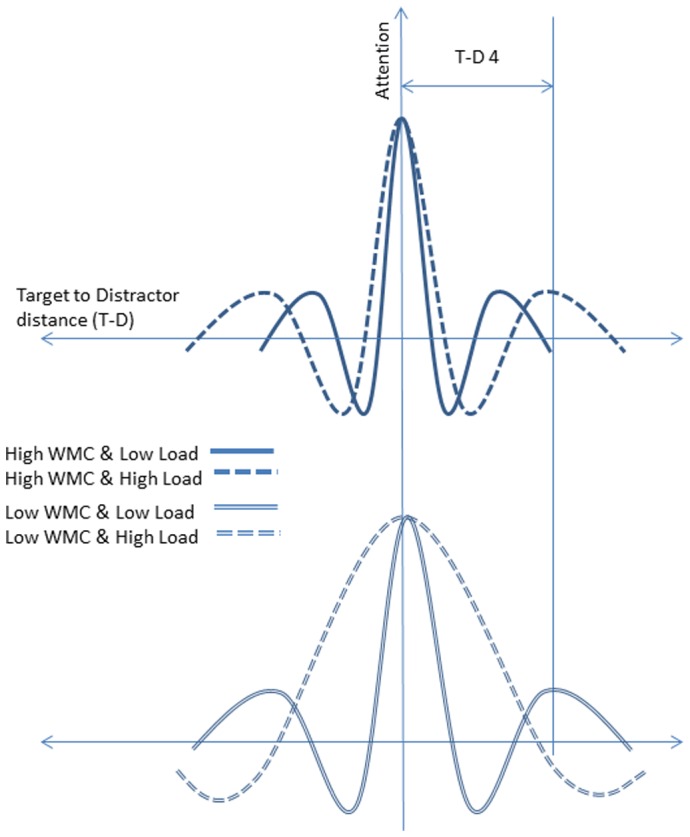
The proposed modulation of the Mexican-hat distribution as a function of WMC and WML. The schematic representation illustrates the proposed dispersion of the Mexican-hat profile as a function of cognitive limitations and also explains the inverted-U pattern of congruency effects recorded at TD 4 in Experiment 1 & 2.

Why spatial attention should show a profile in the shape of a Mexican-hat is a matter of debate, and several explanations, including anatomical, psychological and cognitive models, have been proposed [20]–[23]. Relevant to the current findings, the Mexican-hat profile has recently been shown to be sensitive to manipulations of WML [24]. When congruency effects are measured under either high or low concurrent WML, and plotted as a function of target-to-distractor distance, the order of the attention and suppression zones remains the same under different levels of WML; however the width of each zone is greater under high, compared to low WML. Thus, the Mexican-hat profile becomes spatially dispersed when WML is high. Similar to the effect of WML on the Mexican-hat profile, we propose that a reduction in WMC leads to a similar dispersion of the distribution of the Mexican-hat profile. Moreover, the Mexican-hat will become even more dispersed when both WML and WMC act to reduce WM resources, i.e., under high WML in low WMC individuals (see Figure 3).

The pattern of congruency effects observed in Experiment 1 is in line with the notion that the attentional profile becomes dispersed by both WML and WMC, and that a combination of these factors leads to an even further dispersion of the Mexican-hat profile. With optimal WM availability (under low WML in high WMC individuals), the Mexican-hat profile is predicted to be most constrained, and the fixed distance distractor would have coincided with the outer suppression zone (s2). Consequently, congruency effects would be small in this condition, just like we found (M = 16 ms). As WM resources are reduced, either because of high WML, or because of low WMC, the Mexican-hat profile would become dispersed, and the distractor would coincide with the second attention zone (a2). This would explain the greater congruency effects we observed under high WML in the high WMC group (M = 51 ms), and under low WML in the low WMC group (M = 45 ms). Finally, under high WML in the WMC group, the Mexican-hat profile may become dispersed even further, causing the distractor to coincide with the first suppression zone (s1). This can explain the reduction in congruency effects under high WML in the low WMC group in our results (M = 20 ms).

The notion that the attentional profile may be susceptible to variations in WMC is indirectly supported by previous work showing that there is substantial variation in the distribution of the Mexican-hat between individuals [25], and also that WMC predicts attentional flexibility. For instance, WMC predicts task appropriate discontiguous allocation of attention [7], the speed of constraining attention [26], as well as maintaining a constrained focus of attention for a longer duration [3]. Previous work by Caparos and Linnell [24] has found a dispersion in the profile following manipulations of WML. However when recording the effect of high and low perceptual load on the attentional profiles of high and low WMC groups using perceptual salience-related interference effects they found that the overall amplitude of the low WMC groups' perceptual interference was greater but the two groups' spatial distributions of the Mexican-hat profile did not vary when the level of perceptual load, rather than WML, was manipulated. It remains therefore unclear to what extent individual differences in WMC are in fact associated with different distributions of the Mexican-hat profile as a function of WML.

The explanation of the pattern of congruency effects observed in Experiment 1 as a function of WML and WMC in terms of changes in the spatial dimension of the Mexican-hat profile is of course speculative, as the spatial characteristics of the Mexican-hat profile had to be inferred from the effects of a single distractor that was always presented at the same distance from the target. In Experiment 2 we recorded the profile of spatial attention by measuring congruency effects across four target-to-distractor distances, for low and high WMC individuals during low and high WML. This way, we could map if the attentional profile is indeed modulated as a function of WML and WMC as we hypothesise, and also whether the apparent selective attention improvement in the low WMC group under high WML in Experiment 1 can be explained in terms of the combined effect of these factors on the Mexican-hat profile.

## Experiment 2

### Method

**Participants:** Eighty university students (13 males, mean age = 21.05 years, SD = 4.09) took part in the study. All had reported normal or corrected–to-normal vision, and received course credits or payment for taking part.

**WMC screening:** Aospan scores were obtained in the same way as in Experiment 1.

**Stimuli and procedure:** Stimuli and procedure were the same as in Experiment 1 apart from the following aspects. Firstly, the distractor was presented at one of four possible distances from the target in order to map the distribution of the attentional window. The Mexican-hat distribution has previously been observed across a range of target-to-distractor distances [18], [17], [24]. Experiment 2 aims to map the spatial profile between the target and the distractor position used in Experiment 1, we thus used target-to-distractor distances incrementally closer to the target than the target-to-distractor distance used in Experiment 1. Edge-to-edge target-to-distractor distances of 0.8°, 1.34°, 2.22° and 3.35° were used. Secondly, the distractor was presented either above, below, to the left or to the right of the target (see Figure 1c). Finally, in order to prevent the distractor from appearing too far towards the edge of the screen, we only used the two central target positions this time. The edge-to-edge distance between the target positions was approximately 0.37°. The target was equally likely to appear in any of the two positions, and was presented with a distractor that was equally likely to appear at each of the 16 possible distractor locations. In Experiment 2, a 50 ms duration fixation display preceded each letter display (see Note 1) and was followed by a 200 ms blank screen.

The combination of target identity (2), target position (2), distractor identity (2), target-to-distractor distance (4) and distractor position (4) generated 128 unique displays, each of which was presented once in a block. There were 46 WM trials per block, and two blocks of each load condition. There were 20 WM trials that had two letter identification trials, 16 WM trials with three letter identification trials, and ten WM trials with four letter identification trials. Each experimental block began with one practice trial. Participants completed two practice blocks of each load condition, consisting of three load trials and between 6–12 letter trials per load condition. The experimental session lasted 50–60 minutes.

### Results

**Data Screening:** Data from three participants with below chance accuracy on either the WM or the flanker task were removed from the analysis.

**WMC groups:** From the remaining 77 participants, those with Aospan scores in the upper and lower quartiles were classified as high and low WMC individuals, respectively. This resulted in 22 participants per WMC group (Aospan score: Low Group, M = 12.68, SD = 7.09; High Group, M = 53.86, SD = 7.52).

**WM task:** Mean error rate was computed for participants in each WMC group as a function of WML (Table 1). The errors rates were analysed in a 2×2 ANOVA with WML (low, high) as a within subjects factor and WMC (low, high) as a between subjects factor. Probe error rates were lower under low load (M = 14%) compared to high WML (M = 20%; F(1,42) = 17.75, MSE = 0.004, p<.0001, 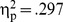). The main effect of WMC group was also reliable, F(1,42) = 8.22, MSE = 0.013, p<.006, 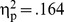. Error rates were higher in the low WMC group (M = 20%) compared to the high WMC group (M = 13%). The interaction between WML and WMC was also reliable, F(1,42) = 7.04, MSE = 0.004, p<.01, 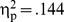. As shown in Table 1, the difference in error rates between low and high WML was greater in the low, compared to the high WMC group.

**Flanker task:** For the analysis of letter identification responses, the same criteria as in Experiment 1 were used to exclude trials. Mean correct RT and error rate were computed for each participant in the two WMC groups as a function of congruency condition, WML, and target-to-distractor distance (Table 3 and 4). From the distractor position nearest to the target to the position furthest away, the four target-to-distractor distances were labelled d1, d2, d3, and d4, respectively. To increase clarity, the analysis was performed on congruency effects, which were calculated by subtracting mean RT in the congruent condition from mean RT in the incongruent condition. Congruency effects in the error rates were calculated by subtracting the mean error rate in the congruent condition from the mean error rate in the incongruent condition.

**Table 3:** Experiment 2: Mean correct reaction times (in milliseconds) on the letter identification task as a function of WML, target to distractor distance, congruency type, and WMC Groups.

		Target-to-distractor distance			
		1	2	3	4
**Low WMC**					
Low WML	Congruent	597 (125)	605 (122)	596 (124)	586 (124)
	Incongruent	654 (140)	624 (137)	632 (140)	627 (138)
	Congruency Effect	57 (54)	10 (63)	36 (47)	41 (45)
High WML	Congruent	607 (105)	600 (131)	613 (139)	607 (119)
	Incongruent	666 (135)	636 (139)	631 (120)	609 (107)
	Congruency Effect	59 (53)	36 (50)	18 (56)	2 (41)
**High WMC**					
Low WML	Congruent	593 (87)	592 (95)	570 (89)	587 (90)
	Incongruent	635 (98)	608 (86)	602 (100)	600 (97)
	Congruency Effect	43 (57)	16 (40)	33 (45)	15 (51)
High WML	Congruent	591 (89)	585 (91)	572 (69)	579 (82)
	Incongruent	633 (88)	594 (79)	596 (78)	612 (94)
	Congruency Effect	42 (78)	9 (38)	24 (51)	33 (34)

Values in brackets are Standard Deviations.

**Table 4:** Experiment 2: Mean error rates on the letter identification task as a function of WML, target to distractor distance, congruency type, and WMC groups.

		Target-distractor distance			
		1	2	3	4
**Low WMC**					
Low WML	Congruent	0.10 (0.09)	0.1 (0.08)	0.09 (0.12)	0.11 (0.11)
	Incongruent	0.16 (0.11)	0.14 (0.12)	0.12 (0.11)	0.11 (0.10)
	Congruency Effect	0.06 (0.09)	0.04 (0.08)	0.03 (0.09)	0.00 (0.07)
High WML	Congruent	0.11 (0.11)	0.1 (0.08)	0.14 (0.09)	0.08 (0.08)
	Incongruent	0.15 (0.10)	0.12 (0.10)	0.12 (0.09)	0.11 (0.08)
	Congruency Effect	0.04 (0.11)	0.02 (0.09)	0.02 (0.08)	0.02 (0.08)
**High WMC**					
Low WML	Congruent	0.08 (0.08)	0.08 (0.08)	0.09 (0.09)	0.10 (0.09)
	Incongruent	0.11 (0.08)	0.10 (0.09)	0.09 (0.09)	0.11 (0.10)
	Congruency Effect	0.03 (0.09)	0.02 (0.09)	0.00 (0.07)	0.02 (0.08)
High WML	Congruent	0.06 (0.07)	0.07 (0.07)	0.07 (0.06)	0.06 (0.09)
	Incongruent	0.12 (0.09)	0.11 (0.10)	0.10 (0.11)	0.08 (0.09)
	Congruency Effect	0.06 (0.09)	0.03 (0.07)	0.03 (0.07)	0.02 (0.08)

Values in brackets are Standard Deviations.

We first checked that the inverted-U pattern of congruency effects observed in Experiment 1 was replicated in Experiment 2. To do this, RT congruency effects at target-to-distractor distance d4 were analysed, which was the distance most similar to that between the target and the distractor in Experiment 1. A 2×2 ANOVA with WML (high, low) as a between and WMC (high, low) as a between subjects factor yielded a significant WML by WMC interaction, F(1,42) = 10.80, MSE = 1698.77, p<.002, 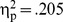 at distractor distance d4. As in Experiment 1, WML had opposing effects on distractibility in the two WMC groups. The congruency effects in the high WMC group increased from 15 ms under low load, to 33 ms under high load (t(21) = 1.599, SEM = 11.55, p = .125), whereas in the low WMC group congruency effects decreased from 41 ms under low load to 2 ms under high load (t(21) = 2.96, SEM = 13.24, p<.007). The main effects of WMC and WML were not reliable (p>.5 in both cases).

We again also ran an analysis including all participants, as a further test of the relationship between the factors of WMC and WML, and distractibility. The 33 participants not included in the initial analysis were added to this analysis. They had intermediate WMC (Aospan Score, M = 33.52, SD = 7.52). In order to test if WMC significantly predicted RT congruency effects in the low and high WM load conditions, we ran a regression analysis with WMC as a predictor, and congruency effect as the predicted variable. The results of the regression indicated that there was a significant inverse relationship between WMC and congruency effects under low WML (*β = *−.714, R_2_ = .051, F(1,75) = 4.07, p = .047). Conversely, under high WML there was a trend towards a positive relationship between WMC and congruency effects (*β = *.702, R_2_ = .037, F(1,75) = 6.09, p = 0.092). This finding again suggests that increasing WML has opposite effects on distractibility in individuals with varying levels of WMC, with reduced interference with increasing WMC when WML is low, and a tendency for greater interference with increasing WMC when WML is high.

Error rates were analysed in the same way as RTs. The 2×2 ANOVA on the error rates revealed no main effects or interaction (p>.5 in all cases). Also in the error rate regression analysis including all participants, there was no significant association between WMC and interference in low WML (R_2_ = .0.006, F(1,75) = .480, p = 0.491) and high WML (R_2_ = .004, F(1,75) = .292, p = .591).

Next, we evaluated the spatial pattern of the congruency effects across the four target-to-distractor distances, again as a function of WML and WMC. RT congruency effects were entered into a 2×4×2 ANOVA, with WML (low, high) and target-to-distractor distance (d1, d2, d3, d4) as within subjects factors, and WMC Group (low, high) as a between subjects factor. The analysis revealed a reliable main effect of target-to-distractor distance, F(3, 40) = 5.33, MSE = 2639.96, p<.003, 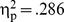. The overall congruency effect was 50 ms at d1, 20 ms at d2, 27 ms at d3, and 23 ms at d4. No other main effects or two-way interactions were reliable (p>.2 for all effects). Crucially however, there was a reliable three-way interaction between WML, target-to-distractor distance and WMC, F(3, 40) = 3.87, MSE = 1995.11, p<.016, 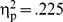, confirming that the spatial profile of congruency effects was significantly different as a function of WMC and WML.

**Figure 4:**
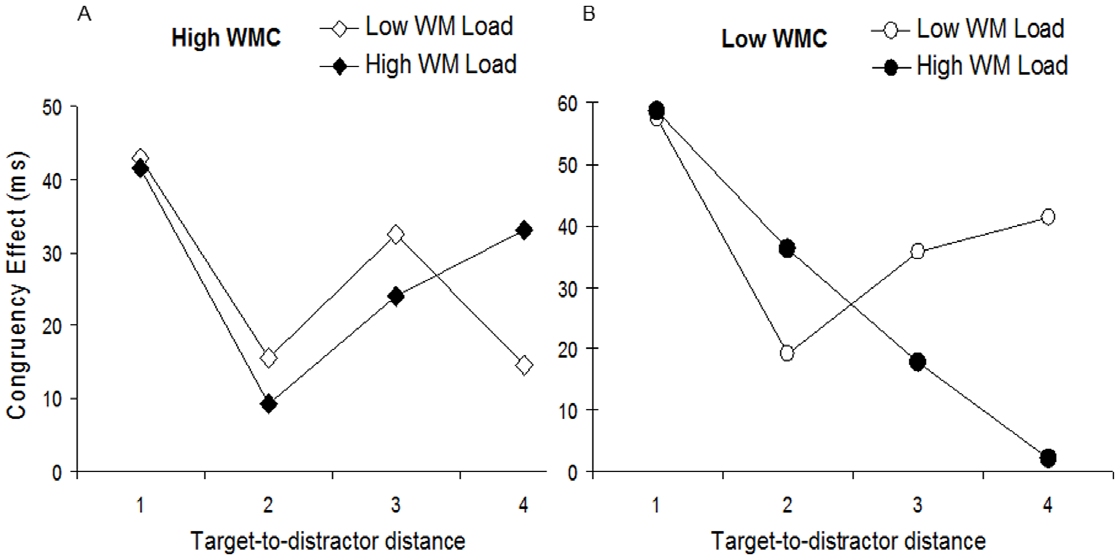
**Experiment 2** **RT congruency effects graph.** Mean congruency as a function of target-to-distractor distance in A) High and B) Low WMC groups under High and Low WML. Note that the typical Mexican-hat profile is evident in High WMC under Low Load, with a relatively strong congruency effect at distance d1 (first attention zone, a1), followed by weaker congruency at distance d2 (first suppression zone, s1), stronger congruency at distance d3 (second attention zone, a2), and finally weaker congruency at distance d4 (peripheral suppression or unattended zone, s2).

In the high WMC group, the Mexican-hat profile was spatially most constrained under low WML (see Figure 4a). There were strong congruency effects at d1 (M = 43 ms) and d3 (M = 33 ms), and much weaker congruency effects at d2 (M = 16 ms) and d4 (M = 15 ms). Thus, all four components of the Mexican-hat profile (a1, a2, s1, s2, respectively) were captured across the target-to-distractor distances in this case. The Mexican-hat profile became more spatially dispersed under high WML in the high WMC group, with only the first three components of the profile occurring across the same target-to-distractor distances. Now, congruency effects were strong at d1 (M = 42 ms) and decreased at d2 (M = 9 ms) but remained high at d3 (M = 24 ms) and d4 (M = 33 ms).

In the low WMC group under low WML, the Mexican-hat profile was more spatially dispersed compared to the high WMC group in the same WML condition (see Figure 4b). Congruency effects were strong at d1 (M = 57 ms), weaker at d2 (M = 10 ms), and then stronger again at d3 (M = 36 ms) and d4 (M = 41 ms). These effects correspond with the first three components of the Mexican-hat profile (a1, s1, a2, respectively). Under high WML load in the low WMC group, congruency effects were strong at d1 (M = 59 ms) and d2 (M = 36 ms), and weaker at d3 (M = 18 ms) and d4 (M = 2 ms). These effects correspond with just the first two components of the Mexican-hat profile (a1, s1, respectively), indicating that the Mexican-hat profile was yet more spatially dispersed in this case.

Congruency effects in the error rates were entered in a similar 2×4×2 ANOVA as the RTs. The only reliable effect was a two-way interaction between WMC and WML, F(1, 42) = 5.83, MSE = 0.004, p<.020, 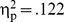. For the high WMC group, mean congruency effects in the error rates increased with WML, whereas in the low WMC group overall congruency effects were smaller under high compared to low load.

### Discussion

To explain the inverted-U shaped function of distractibility effects observed in Experiment 1, we proposed that limits in WM resources hindered the ability to constrain the spatial profile of attention, causing the fixed distance distractor to occur at different sections of the Mexican-hat profile of attention. In Experiment 2, the spatial distribution of attention was mapped in individuals with low or high WMC, and under either low or high WML by measuring the congruency effects from distractors that appeared at varying distances from the target. This allowed a test of the prediction that a limitation in WM resources leads to greater dispersion in the spatial profile of attention when either WMC is low or WML is high, and even more so when both these conditions apply.

In high WMC individuals under low WML, we found that the congruency effects were closely described by the standard Mexican-hat profile [17]–[18]. Congruency effects were better described by a dispersed Mexican-hat profile as WM became less available, either through low WMC or high WML. Most importantly however, when the availability of WM was most compromised, in the low WMC group under high WML, the congruency effects were best described by an even more dispersed Mexican-hat profile. These results replicate recent findings of the effect of WML effect on the spatial distribution of distractor effects [24] (see also Note 2), and extend on these by showing firstly that limitations in WM resources due to individual variations in WMC cause a similar dispersion in the attentional profile, and secondly that the two factors of WMC and WML together have an additive influence on attentional constraining, since the profile became even more dispersed when low WMC and high WML co-occurred.

Given the present results, we can now explain the unexpected inverted- U pattern of congruency effects observed in Experiment 1, which is replicated in Experiment 2 at the target-distractor similar to that in Experiment 1 (i.e. distance d4; see Figure 4). The results support the argument that in Experiment 1, the dispersion of the Mexican-hat profile as a function of differences in WMC and WML caused the distractor to coincide with a different section of the profile in each condition resulting in the inverted-U pattern. In more general terms, the current findings have verified for the first time that depleting WM resources by either individual variations in WMC or imposed external WML reduces the ability to effectively constrain the Mexican-hat profile of attention to relevant information.

## General Discussion

The aim of the current experiments was to investigate the interactive effect of WML and WMC on selective attention. Our findings have shown that congruency effects follow a complex pattern as a function of target-to-distractor distance that is nonetheless remarkably well-described by changes in the Mexican-hat profile as a function of WMC and WML. The combination of low WMC and high WML does not lead to the effect predicted in the introduction, that overall distractibility would be greatest in this situation. This prediction was based on previous evidence that both high WML and low WMC are associated with increased distractibility from a distractor at a fixed target-to-distractor distance [8], [3]. High WML in individuals with low WMC was therefore expected to produce an extreme situation of high distractibility. Our data clearly refute this prediction, as congruency effects in the low WMC group were reduced under high WML. The observed pattern of distractibility as a function of WMC and WML was however perfectly in line with the alternative proposal that the spatial profile of attention is modulated as WM resources become scarce (see Figure 3). Not only did our findings replicate evidence that the Mexican-hat profile becomes more dispersed by high WML [24], in addition, by using representative high and low WMC groups, we show a similar effect in individuals with low WMC, and that the combination of WML and low WMC leads to further dispersing of the Mexican-hat profile.

The current findings shed further light on the mechanism underlying the role of WM in selective attention. It seems that the unavailability of WM affects selective attention, not by modulating the extent to which irrelevant to-be-ignored information is processed per se [8], but instead by changing the spatial profile of attention. The notion that WM limitations lead to less effective constraining of the spatial distribution of attention is compatible with the view that attention has a diffuse default setting, that can be constrained to selectively attend to relevant information [15], [27]. The constraining of attention can be triggered both exogenously and endogenously. For example, the size of the attentional window can be modulated by the size of an exogenous display cue [28]–[30]. In the absence of such exogenous events, as in the current experiments, endogenously controlled attention mechanisms are required to constrain attention. It is well-established that processes under controlled attention draw on capacity limited cognitive resources, and consequently loading or depleting cognitive resources compromises the efficiency by which such processes are executed [31]–[33]. The decline in effective endogenous attentional constraining recorded in the current experiments represents an additional controlled process that is hampered as cognitive resources are limited; the findings are thus consistent with previous findings and theoretical views.

An inability to effectively constrain attention to relevant information as WM resources are depleted not only accounts for the current findings but also provides a plausible mechanism for previously reported increased distractibility with WM limitations. For instance, Lavie et al [8] recorded greater interference from a fixed distance distractor when WML was high, whilst Redick and Engle [3] found low WMC individuals are less able to overcome the influence of a similar fixed distance distractor. In both these cases, the greater influence of the peripheral distractor can be explained by a failure to effectively constrain attention to the relevant target and avoid processing of the distractor when WM resources are limited.

Furthermore, whilst WMC have been reported to predict selective attention efficiency [2], [3], [10], [11], [34], more recent evidence suggests that the effects of WMC are not observed in all selective tasks, but rather are confined to situations that require active adjustment of the attentional settings, such as constraining or restraining of attention [26], [27], [35], [36]. Such findings have led to the proposition that WMC-related differences in selective attention may specifically be driven by individual variations in the adjustability of attention. In the current study, we found that high WMC individuals were indeed better able to adjust their attentional window to task relevant information compared to low WMC individuals, thus providing empirical support for WMC-related variations in the adjustability of visual attention.

The current findings extend those of Caparos and Linnell [24]. When recording the effect of WMC on the attentional profile using a perceptual load manipulation that involved target search for a perceptually salient target (low perceptual load) or non-salient target (high perceptual load), the overall magnitude of the salience-related interference was greater in the low (versus the high) WMC group, but the spatial distributions of the Mexican-hat profile did not vary as a function of perceptual load between groups. The authors speculated that the cognitive manipulation may not have been powerful enough to detect the influence of cognitive limitations (i.e.WMC) in their paradigm. In line with this view, our findings show that when recording the attentional distribution of high and low WMC groups in a cognitively more demanding situation (whilst performing a concurrent WM task), the spatial profiles on the two groups do differ and also that unlike perceptual load, loading working memory does affect the spatial distribution of attention differently as a function of WMC.

Finally, while the current findings sit well within the existing WM and selective attention literature, it is important to acknowledge that they do not speak to situations in which avoiding irrelevant information by effectively constraining attention is not an option, such as when distracting information is not spatially distinct from the relevant target [37], or when targets and distractors are presented successively [38]. Similar to the current findings, in such situations increases in distractibility occur when cognitive resources are limited due to either individual differences in WMC [2], assumed differences in WMC associated with aging [38], [39], or imposed WML [9]. It is hard to explain such findings in terms of greater spatial dispersion of attention, suggesting that WM may also affect the influence of irrelevant information in a manner that does not rely on the adjustment of the attentional window, for instance when selection between perceived relevant and irrelevant information occurs at later stages based on category or response selection [40] may also rely on the availability of WM. The latter effects of cognitive load on distractibility are compatible with load theory of attention [8].

The current empirical work thus offers a mechanism by which variations in selective attention efficiency, either because of individual differences in WMC, or because of variations in concurrent WML, can be explained in terms of their effect on spatial selective attention. We have shown that the counter-intuitive reduction in distractibility in people with low WMC whose WM is highly loaded (compared either to people with low WMC under low WML, or to people with high WMC under high WML) can be explained in terms of differences in the spatial dispersion of the distribution of attention. Although there is extensive previous research to demonstrate that WMC is associated with the efficiency of executive attention [1], the precise mechanism of the association has remained unclear. Our novel findings of the effect of WMC, and the combination of WMC and WML suggest that it is the spatial profile of selective attention that is affected by these factors. WMC has reliable associations with various higher order cognitive functions [41]–[43], as well as general intelligence [44], [45], and these associations may in part be explained by the role of WM in the spatial distribution of attention, and a more general variation in attentional flexibility between low and high WM individuals.

### Note 1

We checked that the results in Experiment 1 were the same for the three fixation intervals used in that study (500 ms, 750 ms and 1000 ms). In neither the RTs nor the error rates for Experiment 1 did any effects vary as a function of fixation interval. In Experiment 2 therefore, we used a fixed fixation interval of 50 ms, followed by a 200 ms blank screen. The shorter duration was chosen in order to reduce trial length in Experiment 2, which included a far greater number of trials than Experiment 1.

### Note 2

The congruency effects as a function of target-to-distractor distance in all participants (n = 77) were examined to assess if previously reported effects of WML on the Mexican profile were replicated [24]. During low WML the congruency effects were highest at d1 (46 ms), then decreased to 26 ms at d2, then increased to 34 ms at d3, and finally decreased again at d4 (18 ms). The pattern of congruency effect was compatible with the Mexican-hat distribution, and indicated that the two attention and two suppression zones of the profile were represented in the measured congruency effects. Moreover, we found that the profile of spatial attention became more dispersed when WML was increased: under high WML, the congruency effect was highest at d1 (42 ms), and decreased at d2 to 28 ms, and continued to decrease at d3 (15 ms). The congruency effect increased after d3, and was 21 ms at d4. The results indicate that only the first three zones of the attentional window are represented in the high WML condition (a1, s1, and a2), whereas as all four zones are represented in the low WML condition, and are thus compatible with a WML related dispersion in the attentional profile.
